# Mathematical modeling for resting state functional connectivity of cortical and sub-cortical networks

**DOI:** 10.1186/1471-2202-14-S1-P101

**Published:** 2013-07-08

**Authors:** Mohit H Adhikari, Alessandra Griffa, Patrick Hagmann, Maurizio Corbetta, Gustavo Deco

**Affiliations:** 1 Theoretical and Computational Neuroscience, Center for Brain and Cognition, University of Pompeu Fabra, Barcelona, 08018, Spain; 2 Signal Processing Laboratory, Ecole Polytechnique Fédérale de Lausanne (EPFL), Lausanne, Switzerland; 3 Department of Radiology, Lausanne University Hospital (CHUV) and University of Lausanne (UNIL), Lausanne, Switzerland; 4 Washington University in St. Louis, School of Medicine, Department of Neurology, Radiology, Anatomy and Neurobiology, St. Louis, USA

## 

Mathematical modeling studies for brain activity at rest have mainly focused on networks of cortical areas. However, cortical areas are connected to sub-cortical areas, such as the thalamus and the hippocampus, whose contribution to the resting state activity cannot be ignored. Further, modeling for diseases such as stroke, in which the focus is usually in a sub-cortical region, requires taking sub-cortical networks into consideration.

Here, we use the structural connectivity (SC) matrix of 83 areas (68 cortical, 15 sub-cortical) from 10 subjects, obtained using diffusion spectrum imaging, to model the activity of these regions. Each node of this network is modeled using the dynamical mean field model of Wong and Wang [1,2] involving a single stochastic differential equation for the population firing rate. In the absence of coupling, each area is in the spontaneous state of low firing rate. Next, we simulate deterministic dynamical equations for the first and second order statistical moments of this stochastic system and obtain simulated functional connectivity (sFC). We then vary three coupling parameters - wc, wsc and wcsc - multiplying the SC strengths between (a) cortical areas, (b) sub-cortical areas and (c) cortical and sub-cortical areas respectively and compare the sFC with the empirical functional connectivity (FC) during resting state. We also calculate the bifurcation diagram of the network as a function of these three parameters.

In this three-dimensional parameter space we obtain the maximum correlation between FC and sFC values for both cortical and sub-cortical areas near the bifurcation surface where the spontaneous state loses stability with higher values of w_c _and w_csc _compared to w_sc_. Interestingly, in this region, while the sFC for cortical areas correlates well with the corresponding SC values, it does not do so for sub-cortical areas. This suggests that the functional connectivity between sub-cortical areas during the resting state is influenced more by their anatomical connections with the cortical areas than between themselves. It also validates our choice of using three coupling parameters instead of just one global coupling parameter. We aim to use this study to model the resting state functional connectivity in patients of stroke.
